# Supplementary figures and images for: A novel cell culture system modeling the SARS-CoV-2 life cycle
Source: PLoS Pathog. 2021 Mar 12;17(3):e1009439. doi: 10.1371/journal.ppat.1009439 (PMC7990224; doi:10.1371/journal.ppat.1009439)

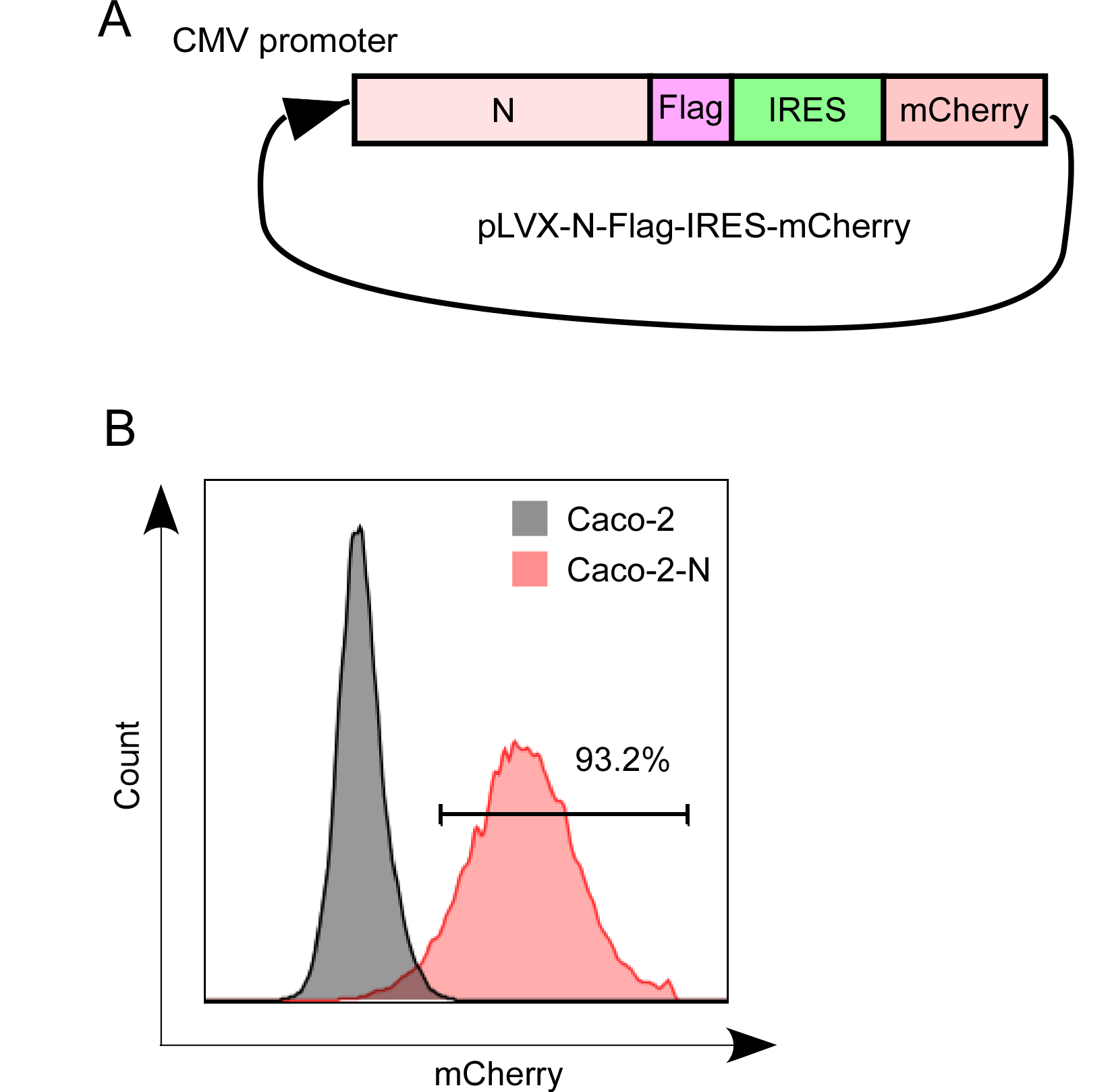

Supplement: S1 Fig — (A) Scheme depicting the bicistronic lentiviral constructs for expressing SARS-CoV-2 N protein with C-terminal Flag tag. (B) Representative flow cytometry plots demonstrating efficient lentivirus transduction. Caco-2 cells were transduced with pLVX-N-Flag-IRES-mCherry or not transduced. Flow cytometric analysis was performed 4 d following transduction to quantify the frequencies of N-expressing cells. The flow cytometry result was representative of one of three independent experiments. (TIF) [file ppat.1009439.s001.tif]

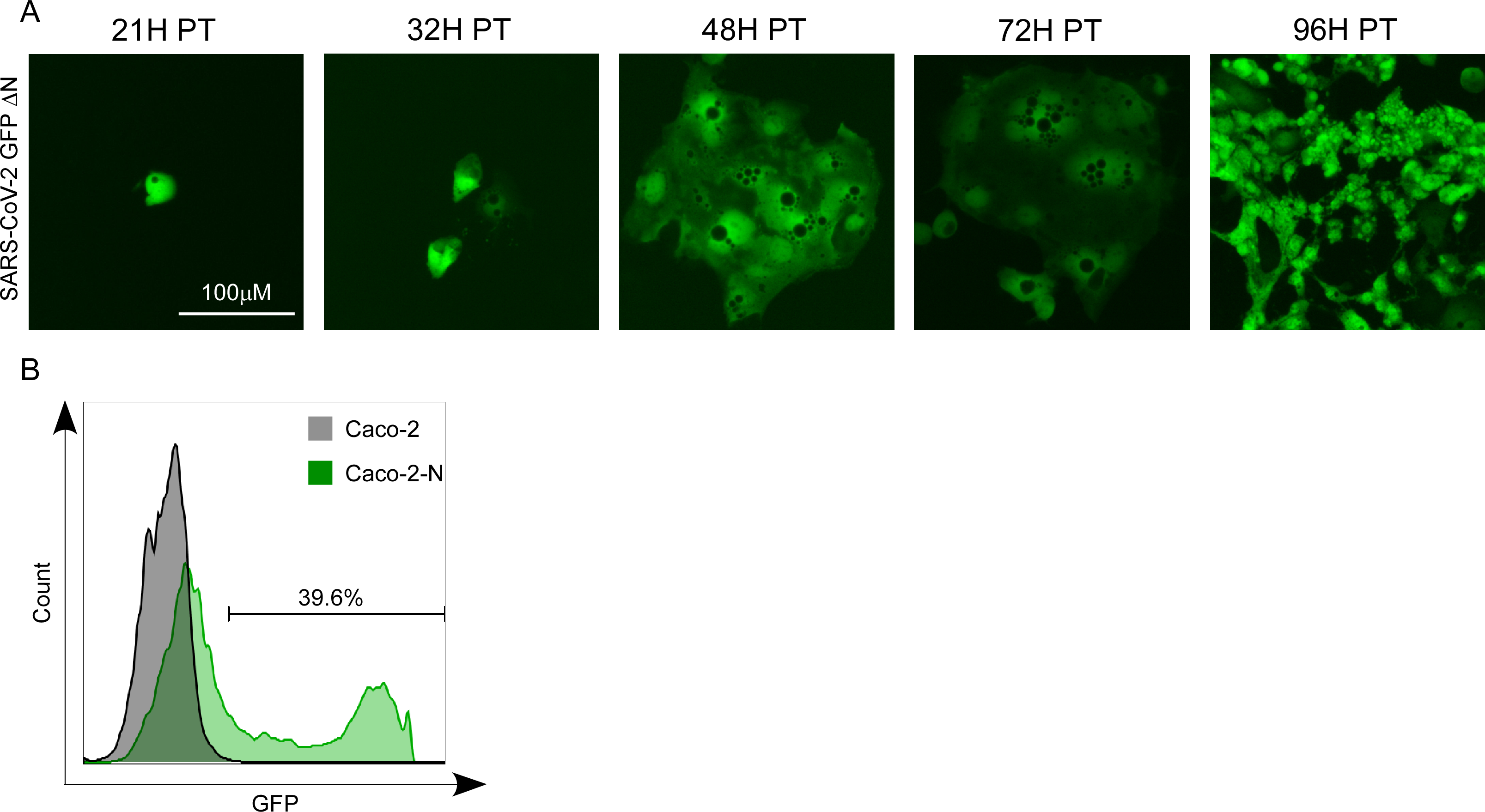

Supplement: S2 Fig — (A) GFP expression in Caco-2-N cells electroporated with SARS-CoV-2 GFP/ΔN RNA. Caco-2-N cells were electroporated with 20 μg of SARS-CoV-2 GFP/ΔN RNA. From 21h-96h p.t., GFP expression in the cells was observed with microscopy. (B) GFP expression was quantified by flowcytometry at 96h post transfection of the RNA. This experiment was representative of three independent experiments. (TIF) [file ppat.1009439.s002.tif]

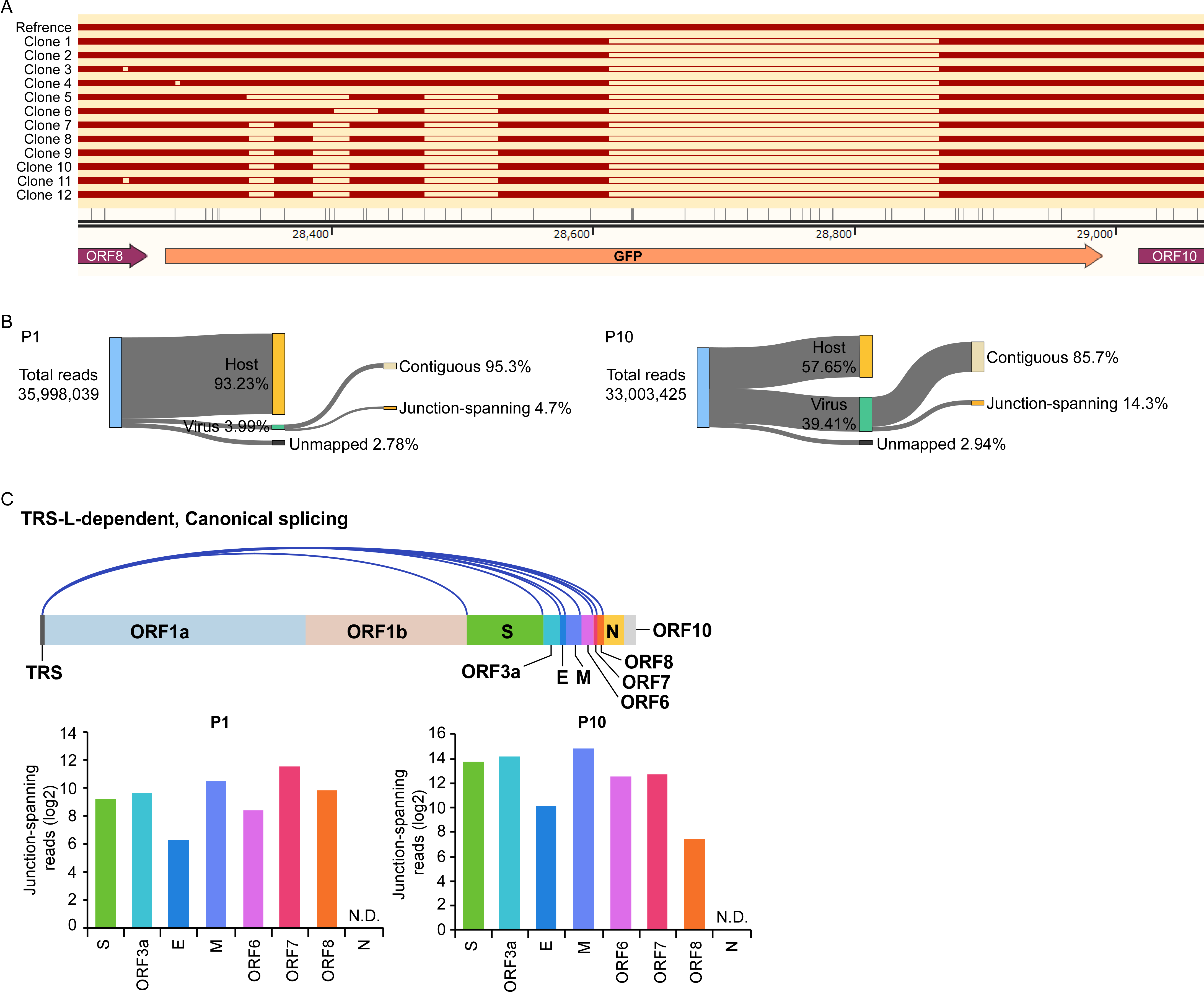

Supplement: S3 Fig — (A) RT-PCR products from P10 virus infected cell passage were cloned into pEASY-Blunt vector, and 12 colonies were randomly chosen for DNA sequences analysis. Multiple deletions were detected in the amplicon. (B) Categories of mapped reads from P1 and P10 virus infected Caco-2-N cells. (C) Canonical discontinuous transcription (top) that is mediated by TRS-L (TRS in the leader) and TRS-B (TRS in the body). Quantification of junction-reads from canonical discontinuous transcripts post P1 and P10 virus infection. (TIF) [file ppat.1009439.s003.tif]

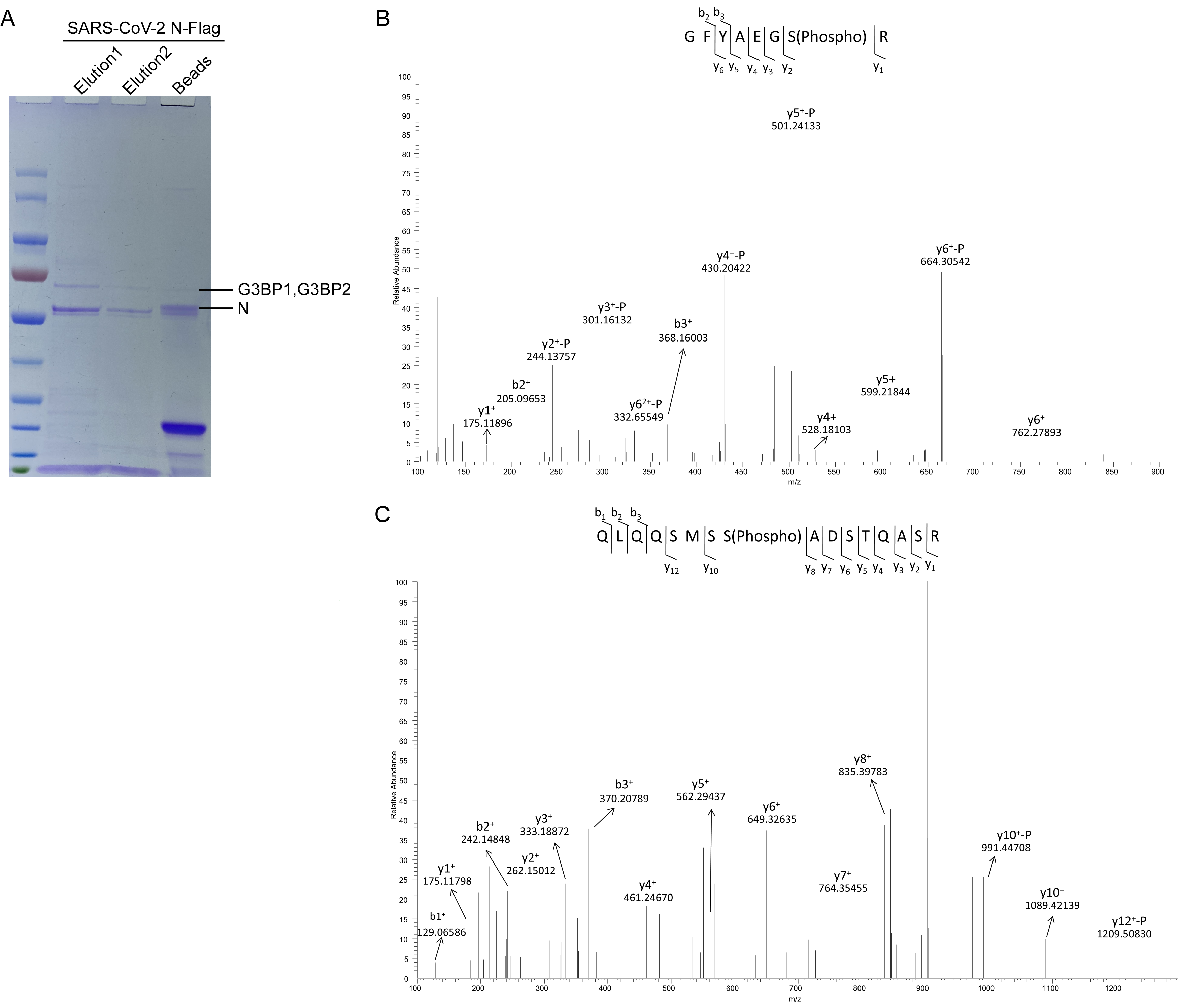

Supplement: S4 Fig — (A) Flag tagged N protein was immunoprecipitated from Caco-2-N cells infected with recombinant SARS-CoV-2 GFP/ΔN trVLP using Flag antibody, and the proteins were analyzed on SDS-PAGE gel. The proteins were visualized by Coomassie blue staining. N, G3BP1 and G3BP2 were labelled. (B) Phosphorylated peptides of N protein derived from Caco-2-N cells. Caco-2-N cells in which N was C-terminal Flag-tagged were collected and cell lysates were immunoprecipitated with anti-Flag coupled beads. Phosphorylated peptides of the immunoprecipitates were analyzed by mass spectrometry. (TIF) [file ppat.1009439.s004.tif]
