# Supplementary material for: A novel cell culture system modeling the SARS-CoV-2 life cycle
Source: PLoS Pathog. 2021 Mar 12;17(3):e1009439. doi: 10.1371/journal.ppat.1009439 (PMC7990224; doi:10.1371/journal.ppat.1009439)
Supplement: Attachment — Submitted filename: Reviewer comments QD_JXH20210224.docx [file ppat.1009439.s007.docx]

**Responses to Reviewers**

**Part I - Summary**

Reviewer #1: In this report, Ju et al., generate and characterise a novel model of SARS-CoV-2 infection, which could be used outside of BSL3 conditions. Through the use of reverse genetics, the team successfully replaced the essential N gene with a fluorescent reporter and stocks generated by infection of cells expression N in trans, complementing the deleted N. To further extend the biosafety of this reagent, Ju et al., exploit the protein splicing intein system to split the in trans N to two genetic fragments, reducing likelihood of recombination and generation of fully infectious SARS-COV-2 outside BSL3 conditions. The team also carry out extensive passaging and sequencing to identify potential changes yielding infectious virus but none were detected. Finally, Ju et al., use this safer reporter system to probe the biology of SARS-CoV-2 N and identify novel antiviral compounds against SARS-CoV-2. This work is an important and timely report on a reagent that would be helpful in our understanding of SARS-CoV-2 infection and identification of potential therapeutic interventions.

*Authors’ response: Thanks for reviewer’s positive comments.*

Reviewer #2: In this manuscript, Ju et al., developed an N gene-based system to produce a transcription/replication competent SARS-CoV-2 virus-like particles in which the N gene is replaced by the GFP coding sequence. In addition, authors utilized a protein splicing technique to express a functional SARS-CoV-2 N protein through trans-splicing of two independently expressed N subunits. Thus the biosafety of using this VLP is further reduced and can be performed under a BSL-2 condition. The N-complemented VLPs recapitulated several properties of the authentic virus, such as the transcriptome profiling, receptor binding and neutralization ability, and drug sensitivity to known antivirals of SARS-CoV-2. Moreover, several novel antivirals were discovered through the high-throughput screening using the N-complemented VLPs. Overall, the system is well designed and provides a convening way to screen potential antivirals of SARS-CoV-2 in a BSL-2 laboratory.

*Authors’ response: Thanks for reviewer’s positive comments.*

**Part II – Major Issues: Key Experiments Required for Acceptance**

Reviewer #1: N/A

Reviewer #2: The manuscript is well organized and phrased, and is recommended to be published if several points listed below can be addressed.

1. Figure 1A: The scheme of the viral genome is incorrect. The 3' end contains poly(A) rather than poly(T).

*Authors’ response: We corrected this in the revised Fig 1A.*

2. line 187-188: Although ORF6, ORF7, and ORF8 genes were lost after 10 passages, the importance of these accessary proteins in viral infection is still unknown. Particularly, the experiments were conducted in a highly permissive cell line. It's better to claim that these proteins might be dispensable for SARS-CoV-2 after its adaptation into certain cell lines.

*Authors’ response: Thanks for the reviewer’s suggestion, and we revised it in the revised manuscript accordingly (Page 9, line 213-215, the version with tracked changes).*

3. line 228-230: The trans-splicing design is smart and the result is intriguingly. However, the introduced G212C mutation is close to R203K/G204R substitutions carried by the recent strains. The possible impact of G212C on the N protein bearing R203K/G204R should be considered and discussed.

*Authors’ response: Thanks for the reviewer’s suggestion, and we revised it in the revised manuscript accordingly (Page 10-11, line 259-261, the version with tracked changes).*

4. line 53: Several vaccines are available by Feb 2021. The statement is better to be updated.

*Authors’ response: We revised it in the revised manuscript accordingly (Page 4, line 72-76, the version with tracked changes).*

5. line 69: ....response and....

*Authors’ response: Sorry for this typo, and we revised it (Page 4, line 92, the version with tracked changes).*

6. line 235: structural proteins "and" the primary....

*Authors’ response: We revised it (Page 11, line 264, the version with tracked changes).*

**Part III – Minor Issues: Editorial and Data Presentation Modifications**

Reviewer #1: I have a few minor concerns surrounding presentation and discussion. The authors could pay particular attention to questions surrounding biosafety of the work carried out and any proposed changes to biosafety for SARS-CoV-2 work using this BSL2-compatible system (points 10 and 13).

*Authors’ response: Thanks for the reviewer’s comments and we answer reviewer’s question point-by-point as below.*

Abstract:

1. update first line (and intro) regarding no antivirals and treatments when a number of therapies (e/g dexamethasone) and vaccines (e.g. Moderna mRNA) have been licensed across the world.

*Authors’ response: We revised it in the revised manuscript accordingly (Page 2, line 24, the version with tracked changes).*

2. Should be BSL-3 (check for minor errors, BLS3)

*Authors’ response: Sorry for this typo, and we revised it (Page 2, line 25, the version with tracked changes).*

3. Is the reporter is GFP or enhanced GFP? This should be clarified in the methods

*Authors’ response: The reporter is GFP, and we apology for any confusion if there is. We clarified this in the methods section (Page 20, line 485-486, the version with tracked changes).*

Results:

4. How did you divide the genome up into cDNA fragments? What logic to decide where the junctions are?

*Authors’ response: We designed the genome according to the presence of BsaI or BsmBI restriction enzyme sites and the convenience of manipulation of the reporter. As described in the Methods, we used the BsaI or BsmBI restriction enzyme to generate specific sticky ends in this system. There are two BsaI restriction enzyme cut sites (~17967nt and 24096nt loci) and one BsmBI cut site (24102nt loci) in SARS-CoV-2 genome, thus we took advantage of this to design the region between 17967nt and 24096nt as fragment C which were digested by BsaI to have the specific sticky ends. We further separated the region from 1nt to 17967nt into two fragments of similar length (A and B, 9.2K or 8.7K respectively, both A and B were digested by BsaI). D fragment is from 24096nt to the beginning of N gene, with 4.1k in length, digested by BsmBI. The fragment carrying reporter gene and the remaining viral genome was designed as another independent fragment E with 1.7k in length digested by BsaI, which are easily manipulated for changes of reporters by molecular cloning approach. The design could be modified accordingly if other restriction enzymes were used or other issued were considered. Thus, we think that the performance of SARS-CoV-2 trVLP system is not highly dependent on the strategy of the dividing viral genome.*

5. 'T7 promotor' should read T7 DNA-dep RNA polymerase promotor (at least once during definition)

*Authors’ response: We followed the reviewer’s suggestion to revise it (Page 6, line 134-135, the version with tracked changes).*

6. What is the rationale for using Caco-2 cells over Vero derivatives?

*Authors’ response: Thanks for the reviewer’s important question. We would like to clarify that this system definitely works in Vero E6 cells and the data is not shown in the manuscript due to the limited space. As reviewer known, Vero cells are African green monkey cells, and Caco-2 cells are human derived cells. We would like to apply this system for virus-host interaction, the reagents such as antibodies, siRNA, shRNA, DNA primers etc. can be easily available for human derived cells. In addition, this system could be used to evaluate and discover the antiviral agents against SARS-CoV-2 infection, however, some antivirals may exert antiviral effect in a species-dependent manner, and especially if the drug’s target is the host protein. Thus, we believed that the results obtained from human cells are more human and medical relevant, we used the Caco-2 cells for all the studies in this manuscript.*

7. Can the authors speculate on the reasoning why the GFP gene was lost?

*Authors’ response: SARS-CoV-2 has evolved very fast as evidence by the emergence of many variants which challenge the vaccine development. The inserted GFP gene is not required for SARS-CoV-2 life cycle and it has no selection pressure for the virus. Thus, we think that virus will lose the GFP gene if it was culture for long time.*

8. FLAG tag should be capitalised throughout (or at least keep it one style or the other for consistency)

*Authors’ response: Thanks for the reviewer’s suggestion and we revised it accordingly.*

Discussion:

9. The discussion around proteomic interaction partners would be helped by reference to the earlier literature on other coronaviruses, e.g. https://www.ncbi.nlm.nih.gov/pmc/articles/PMC3754094/ Emmott et al., 2013 (which include description of N/G3BP binding)

*Authors’ response: We followed the reviewer’s suggestion to cite the earlier study in the revised manuscript as reference 42 (Page 17, line 419, the version with tracked changes).*

10. Additionally, the discussion would be helped (and the biosafety implications furthered) with greater conversation surrounding the potential for the reporter to recombine with infectious virus in BSL2 settings. The reviewers that this is likely very rare but is of potential importance if used widely. Have the authors attempting co-infection and recombination analysis with cells expressing the reporter virus with WT SARS-CoV-2 or other related beta coronaviruses? If the authors do not think this an issue they should say in the discussion.

*Authors’ response: Thanks for reviewer’s question. We added more discussion about this point in the Discussion (Page 16, line 395-399).*

Legends/methods:

11. Why was ACE2 not observed in Vero cell lysates by your western blot? Is this antibody specific to human? Or is it simply an expression level issue?

*Authors’ response: The ACE2 antibody (10108-T24, Sino Biological) is specific to human ACE2 according to the instruction (*[*https://cn.sinobiological.com/antibodies/ace2-10108-t24*](https://cn.sinobiological.com/antibodies/ace2-10108-t24)*). Thus, the specificity of the antibody of ACE2 is one of the reasons for this.*

12. Were the transduced lines selected using an antibiotic like puromycin? I don't see mention of that here but perhaps none was carried out.

*Authors’ response: We did not use any antibiotics for the transduced cell line. We showed the transduction of viral N gene in the S1 Fig, and N gene was cloned into lentiviral vector pLVX-IRES-mCherry. As shown in S1B Fig, the transduction efficiency can reach 93% and the generation of the Caco-2-N cells were briefly described in page 7, line 148-151 (version with tracked changes)*

13. Can the authors update the methods describing the level Biosafety of each kind of experiments were performed at. I.e at what stage if any did they move out of the BSL3 (if at all)?

*Authors’ response: We followed reviewer’s suggestion to describe the level of biosafety of experiments in this study. Our Institutional Biosafety Committee (IBC) approved its use in BSL-2 if the Caco-2-N ^intein^ was used for this system and the virus should be regenerated (protocol in Fig1A) after passage 8. We updated this in the Materials and Method section in the revised manuscript (Page 24-25, line 615-622, the version with tracked changes).*

14. Do the authors plan on depositing these reagents in an open repository or how best to request such valuable research reagents? It would be nice if this were addressed.

*Authors’ response: Thanks for reviewer’s suggestion. We would like to share those reagents with the scientific community, and we did not have the well-established open repository in China. The corresponding author claimed the availability of those reagents in the Materials and Methods section in the revised manuscript (Page 20, line 502-503, the version with tracked changes).*

Reviewer #2: (No Response)
